# Differences in energy source storage in eye stalks between two species of stalk-eyed flies, *Sphyracephala detrahens* and *Cyrtodiopsis dalmanni*

**DOI:** 10.1038/s41598-022-13887-7

**Published:** 2022-06-15

**Authors:** Aoi Miki, Risa Fukuda, Koji Takeda, Ayano Moriya, Yoshitaka Kamimura, Chow-Yang Lee, Takashi Adachi-Yamada

**Affiliations:** 1 Department of Life Science, Faculty of Science, Gakushuin University, 1-5-1 Mejiro, Toshima-ku, Tokyo, 171-8588 Japan; 2 Department of Biology, Keio University, 4-1-1 Hiyoshi, Kohoku-ku, Yokohama, 223-8521 Japan; 3 Urban Entomology Laboratory, Vector Control Research Unit, School of Biological Sciences, Universiti Sains Malaysia, 11800 Penang, Malaysia; 4 Present Address: Department of Entomology, University of California, 900 University Avenue, Riverside, CA 92521 USA

**Keywords:** Sexual selection, Autophagy

## Abstract

Some diopsid flies have sexually dimorphic eye stalks that are assumed to require considerable nutrition for growth but are advantageous in competition and courtship. According to the handicap theory, the eye span in some dimorphic species serves as a reliable signal of individual quality to an opponent. However, it is not well understood how well eye span represents energy source storage. In this study, we focused on two species: *Sphyracephala detrahens*, which has weak dimorphism, and *Cyrtodiopsis dalmanni*, which has moderate dimorphism. We found that the eye stalks of the former species contained more fat bodies than those of the latter species. When the flies were starved, the fat body cells in the eye stalks underwent autophagy. A strong positive correlation was consistently found between eye span and starvation tolerance for *S. detrahens*, while a weak correlation was found for *C. dalmanni*. Furthermore, starvation decreased the contest winning rate between *S. detrahens* pairs with similar eye spans. These findings suggest that the presentation of resource holding potential may be larger than the actual storage ability and that the fidelity of nutritional storage signaling varies; the signal presented by *S. detrahens* is more reliable than that presented by *C. dalmanni*.

## Introduction

Many animal taxa exhibit exaggerated sex-specific traits, such as peacock tail feathers, deer horns, and fish nuptial coloration. The main functions of these characteristics are believed to be related to contests or courtship^1,2^. One of the explanations of why such exaggerated traits have developed over time is the handicap theory^3,4^, which states that the development of exaggerated traits is a handicap for the individual and that the ability to bear such a characteristic reflects the high quality, such as nutritional status, of the individual. If the characteristic is associated with genetic factors, it will spread throughout the population by adaptive natural selection^1,3,5–7^. The handicap theory predicts that possession of highly exaggerated traits reveals a higher survivability, which may also positively affect the fitness of partners and descendants. In fact, many exaggerated traits show positive body size allometry, which can be obtained by ingesting a large amount of nutrients; trait exaggeration is an unrealizable handicap for individuals with poor nutrition^8–10^. However, if much of the energy obtained during the growth stage was consumed during the development of exaggerated traits, an individual would have less energy even though it exhibits exaggerated traits conducive to mating, and the degree of trait exaggeration would not reflect fertility. Therefore, the exaggerated trait displayed by an individual may not always accurately indicate the energy holding status at the time of courtship, but this discrepancy is not well studied.

In so-called stalk-eyed flies (Diptera: Diopsidae: Sphyracephalinae + Diopsinae), compound eyes protrude from the head due to a significantly elongated eye stalk. In some species, eye span is sexually dimorphic; that is, the allometric slope of eye stalks versus body length in males are steeper than that in females in dimorphic species^11–14^. However, many diopsid species are sexually monomorphic, and dimorphisms have evolved polyphyletically in a complicated manner in each of the two subfamilies (Sphyracephalinae and Diopsinae)^13–18^. Stronger sexual dimorphisms are seen in species with long, thin eye stalks and are often associated with mate choice as well as contest outcomes^19–22^. In this study, we used two species belonging to the two subfamilies because both were easily accessible materials for us. One species is *Sphyracephala detrahens* (Walker, 1860), which has weak sexual dimorphism of its short and stout eye stalks. This species engages in ritualized contests in which individuals with longer eye spans benefit irrespective of male–male, female–female, or male–female combinations^23^. Males with a long eye span can mate repeatedly, and females with a long eye span are frequently chosen by males^21,23^. The other species is *Cyrtodiopsis dalmanni* (Wiedemann, 1830), which exhibits moderate sexual dimorphism of long and thin eye stalks^12,13^. *C. dalmanni* is historically the most employed species in behavioral studies of contests and courtship. Males engage in ritualized contests influenced by eye span, and males with longer eye spans are more likely to be chosen by females^20,21,24^. Because these two species show distinct differences in mode of contest, courtship and degree of sexual dimorphism, it was interesting to compare their eye-stalk functions.

Whether the handicap theory can be applied to these stalk-eyed flies has been examined from various aspects. For example, individuals raised in a more nutrient-rich environment develop larger eye stalks^23,25,26^, which can handicap flight ability after adult emergence^27,28^. Moreover, both male and female individuals with longer eye spans have higher fertility^23,29–31^. Development of the eye stalk is influenced by not only environmental factors but also genetic factors^32–34^. In interindividual contests of stalk-eyed flies, individuals with a longer eye span are likely to win^12,20,23^. If this result is explained by the handicap theory, eye span should correlate to some extent with the energy consumption potential during the contest^4,7,35^. It is known that higher nutritional intake during the larval period results in the development of larger eye stalks^23,25,26^, but it is unknown whether the ingested nutrients are retained in adults. That is, the relationship between energy stores during contests and eye stalk sizes is unclear.

In this study, we focused on the presence of fat body tissue inside the thick eye stalk of *S. detrahens*. Fat bodies are an energy source, and it was found that the larger the eye stalk is, the greater its storage capacity. However, between two individuals with similar eye spans, which had a nearly equivalent influence on the contest outcome, the amount of energy available during the contest affected the outcome of the contest. This indicates that the magnitude of the resource holding potential represented by eye span may exceed the actual energy storage ability of eye stalks. On the other hand, *C. dalmanni* has limited fat body tissue in its eye stalks, and the eye span of this species is not strongly related to its current energy storage capacity. These findings suggest the presence of cheaters in the ritualized contests of stalk-eyed flies: we hereafter express the individuals who possess an exaggerated ornament relative to their resource holding potential or actual fighting ability at the time of contest or mate choice as cheaters. The proportion of cheaters varies among species, as *S. detrahens* had a lower prevalence of cheaters than *C. dalmanni*, with longer eye stalks functioning as a target of precopulatory female choice as well as in ritualized contests for territories. We demonstrate the differences in the role of eye span between these two species.

## Results

### Large fat body presence in the eye stalks of *S. detrahens* but not in those of *C. dalmanni*

**Figure 1:**
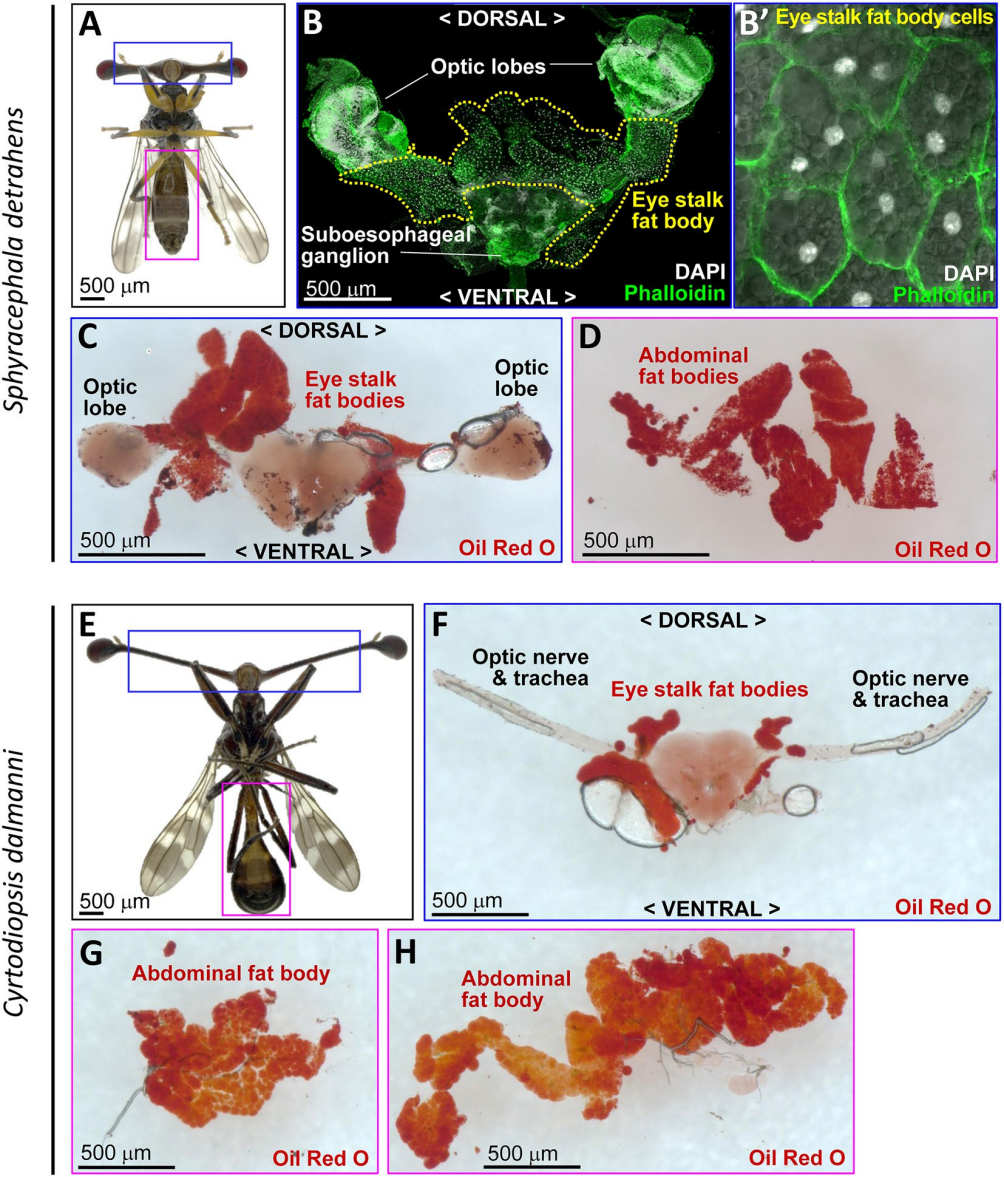
Difference in the volume of fat bodies in eye stalks between two stalk-eyed fly species: *S. detrahens* and *C. dalmanni*. (**A**–**D**) *S. detrahens.* (**A**) Ventral view of an adult male. (**B**) Eye stalk fat body (outlined by the yellow dotted line) present along the optic nerve bundle dissected from the blue-boxed region in (**A**). White: DAPI showing nuclei, green: phalloidin showing filamentous actin. (**B**′) High magnification image of binucleate cells in the eye stalk fat body merged with a differential interference image showing multiple lipid droplets in the cytoplasm. Phalloidin-stained filamentous actin-associated plasma membranes. (**C**) Eye stalk fat body stained with oil red O (brownish-red). (**D**) Abdominal fat body dissected from the magenta-boxed region in A and stained with oil red O. (**E**–**H**) *C. dalmanni.* (**E**) Ventral view of an adult male. (**F**) Eye stalk fat body dissected from the blue-boxed region in E and stained with oil red O. (**G**,**H**) Small and large examples of an abdominal fat body dissected from the magenta-boxed region in E and stained with oil red O.

Consistent differences in the external morphologies of the eye stalks were observed between *S. detrahens* and *C. dalmanni*; the former has thick and short eye stalks, while the latter has thin and long eye stalks (Fig. 1A,E). To determine whether this difference in external morphology was accompanied by a difference in internal morphology, we dissected the eye stalks of the two species. Flat, fat body-like, white tissue was widely distributed in the eye stalks of *S. detrahens* along the trachea and optic nerve (Fig. 1B). Magnified observation of the cells revealed a large number of large vesicles within the cells that were thought to be lipid droplets (Fig. 1B′), which were stained with triglyceride-stainable oil red O, and these organs were considered to be fat body tissue (Fig. 1C). In the eye stalks of *C. dalmanni*, small amounts of similar fat body tissue were present at the base of the eye stalks (Fig. 1F). In *S. detrahens*, the amount of fat body tissue in the eye stalks was almost equal to the amount of abdominal fat body tissue (Fig. 1D). In *C. dalmanni*, the amount of abdominal fat body tissue varied among individuals, but it was always more than the amount of fat body tissue in the eye stalks (Fig. 1G,H). Based on these observations, the thick and short eye stalks of *S. detrahens* are clearly different from the thin and long eye stalks of *C. dalmanni* in terms of fat body development.

### Starvation induces autophagy in both eye stalk and abdominal fat body cells

**Figure 2:**
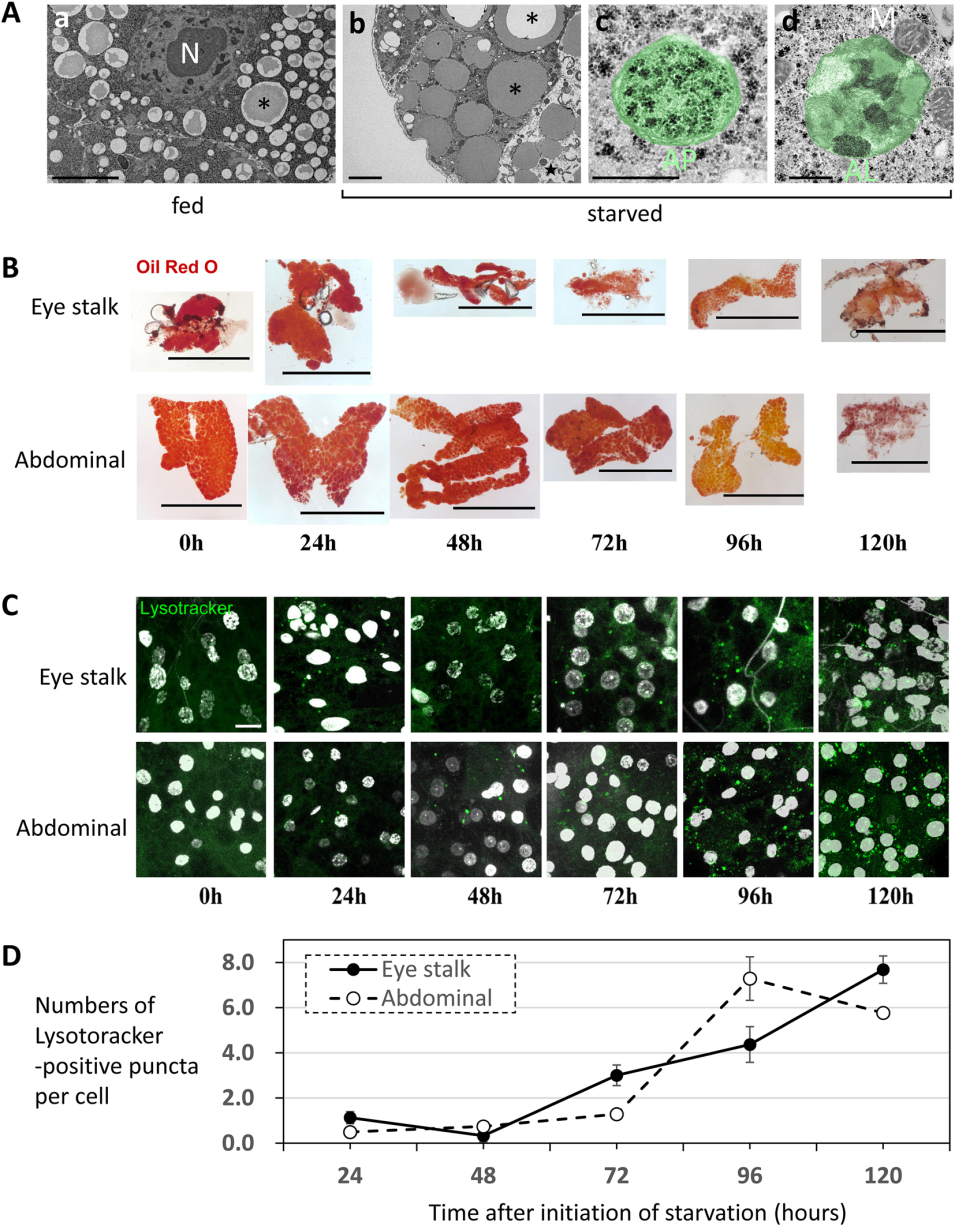
Fat body cells in the eye stalk and abdomen underwent equal autophagy of cytoplasmic components in response to starvation. (**A**) TEM of eye stalk fat body cells. (a) Cells derived from fed individuals. The electron-light lipid component was attached to the inner surface of the lipid droplet membrane in most cases (asterisk). N denotes the nucleus. (b–d) Cells derived from individuals starved for 48 h. (b) In lipid droplets, the electron-light lipid component is completely degraded or detached from the inner surface of the lipid droplet membrane (asterisks). Some cells show a clear cytoplasm (star), suggesting the occurrence of autophagic cell death. (**c**) A representative autophagosome (AP, highlighted with the green pseudocolor) containing glycogen granules that are normally present in the cytoplasm of fat body cells. (d) A representative autolysosome (AL, highlighted with the green pseudocolor) with a multilayered membrane containing organelle debris. A mitochondrion (M) is attached to the autolysosome. Scale bars: 5 μm (a,b), 0.5 μm (c,d). (**B**) Decrease in oil red O staining (brownish-red) in the fat bodies excised from the eye stalk and abdomen in response to starvation. Scale bars: 0.5 mm. (**C**) Increases in the numbers of lysosomes and autolysosomes (green puncta) stained with LysoTracker in fat body cells excised from the eye stalk and abdomen in response to starvation. Scale bar: 10 μm. (**D**) Time course of the number of LysoTracker-positive puncta per cell after the initiation of starvation. All of the data in (**A**–**D**) were derived from female *S. detrahens*. The times in (**B**–**D**) indicate hours after the initiation of starvation.

Fat body cells comprehensively store energy from sources such as sugars and fat for survival, combat, and reproduction^36–38^. If the eye stalk fat body, as well as the abdominal fat body, supplies energy for such activities, nutritional decomposition and autophagy during starvation are expected^39–41^; therefore, we examined these processes in *S. detrahens*. Transmission electron microscopy (TEM) revealed that the starvation of well-fed flies for 48 h, with exposure to only water, resulted in the consumption of fat in the lipid droplets of fat body cells in the eye stalks (Fig. 2Aa,b). In fat body cells, autophagosomes containing glycogen granules (Fig. 2Ac) and autolysosomes responsible for the digestion of organelles (Fig. 2Ad), which are present during autophagy, were observed. Cell death, which is thought to be dependent on autophagy, was also observed (Fig. 2Ab). Observing the fat content in the entire fat body by oil red O staining revealed that the fat content decreased with prolonged starvation (Fig. 2B). In addition, lysosomes and autolysosomes, which are known to increase in quantity during autophagy, increased over time during starvation (Fig. 2C,D). All of these changes were similar between the eye stalks and abdominal fat bodies. These results suggest that the eye stalk fat body, much like the abdominal fat body, stores energy that can be utilized during periods of starvation.

### Starvation tolerance and eye span are strongly correlated in *S. detrahens* but weakly correlated in *C. dalmanni*

**Figure 3:**
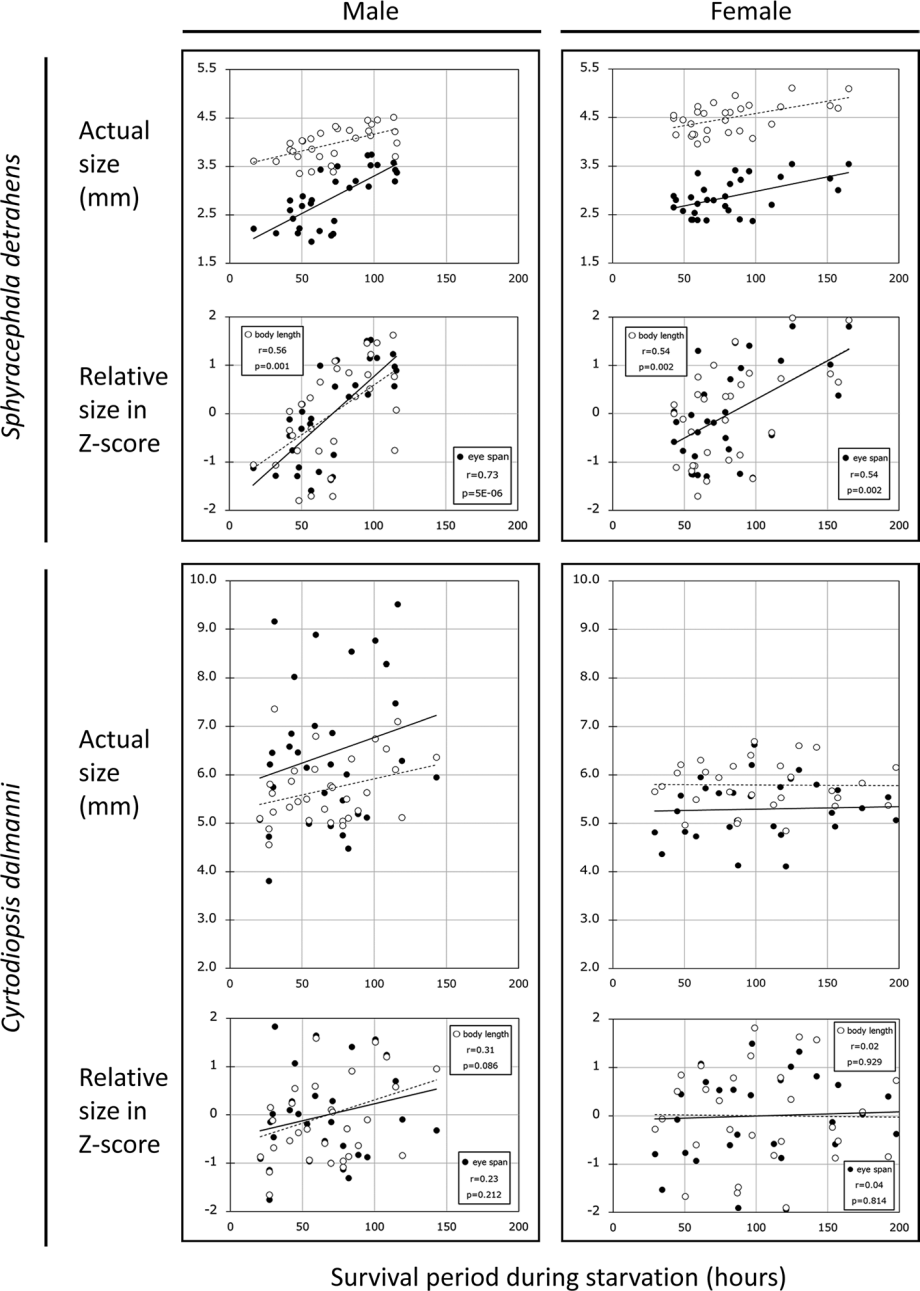
Relationship between starvation tolerance and trait size in both *S. detrahens* and *C. dalmanni*. In *S. detrahens* and *C. dalmanni*, the survival periods of both sexes after the initiation of starvation were measured, and their relationships with eye span (closed circles with solid approximation lines) and body length (open circles with broken approximation lines) are shown (actual size is shown the in upper and Z-scores in the lower graphs, respectively) in each dataset (enclosed by rectangles). The approximation lines were created by ordinary least squares. Both the correlation coefficients (r) and *P* values (p) were the same between the actual size and Z-scores.

The relationship between the amount of energy retained by adults (that is, the number of days of starvation tolerance) and eye span was investigated in males and females of *S. detrahens* and *C. dalmanni*. In male *S. detrahens* (upper left of Fig. 3), starvation tolerance clearly showed a strong positive correlation with eye span (*r* = 0.73, *P* = 5 × 10^–6^); there was also a strong correlation with body length (*r* = 0.56, *P* = 0.001), although it was not as strong as that with eye span. Since there were differences in the means and variances of the measured values between eye span and body length, it is not meaningful to compare the two in the same graphical analysis; thus, both values are represented by relative values (Z-scores) based on their respective means and standard deviations, and the graphs are presented below the graphs for actual size in each data set in Fig. 3. This conversion visually revealed that both eye span and body length correlate with starvation tolerance in almost the same manner, but it can be seen that eye span functions as a more reliable signal than body length. On the other hand, in females, shown in the upper right of Fig. 3, both the correlation between eye span and starvation tolerance (*r* = 0.54, *P* = 0.002) and the correlation between body length and starvation tolerance (*r* = 0.54, *P* = 0.002) were significant, and the contributions of eye span and body length were comparable, although neither were as strong as those of males. The results of *C. dalmanni* greatly contrasted with those of *S. detrahens*. In male *C. dalmanni*, there was a weak positive correlation between body length and starvation tolerance (*r* = 0.31, *P* = 0.086) and a poor correlation between eye span and starvation tolerance (*r* = 0.23, *P* = 0.212), indicating that eye span does not appear to provide a reliable signal of current energy stores. This tendency was even more pronounced in females, in which the correlation between eye span and starvation tolerance (*r* = 0.04, *P* = 0.814) and the correlation between body length and starvation tolerance (*r* = 0.02, *P* = 0.929) were very low. This suggests that neither eye span nor body length represents the current energy stores of females; that is, this trait cannot be used as a signal. In fact, it is known that eye span does not affect the outcomes of contests between female *C. dalmanni*^42,43^.

### Starvation affects the winning rate of contests between individuals with similar eye spans in *S. detrahens*

**Figure 4:**
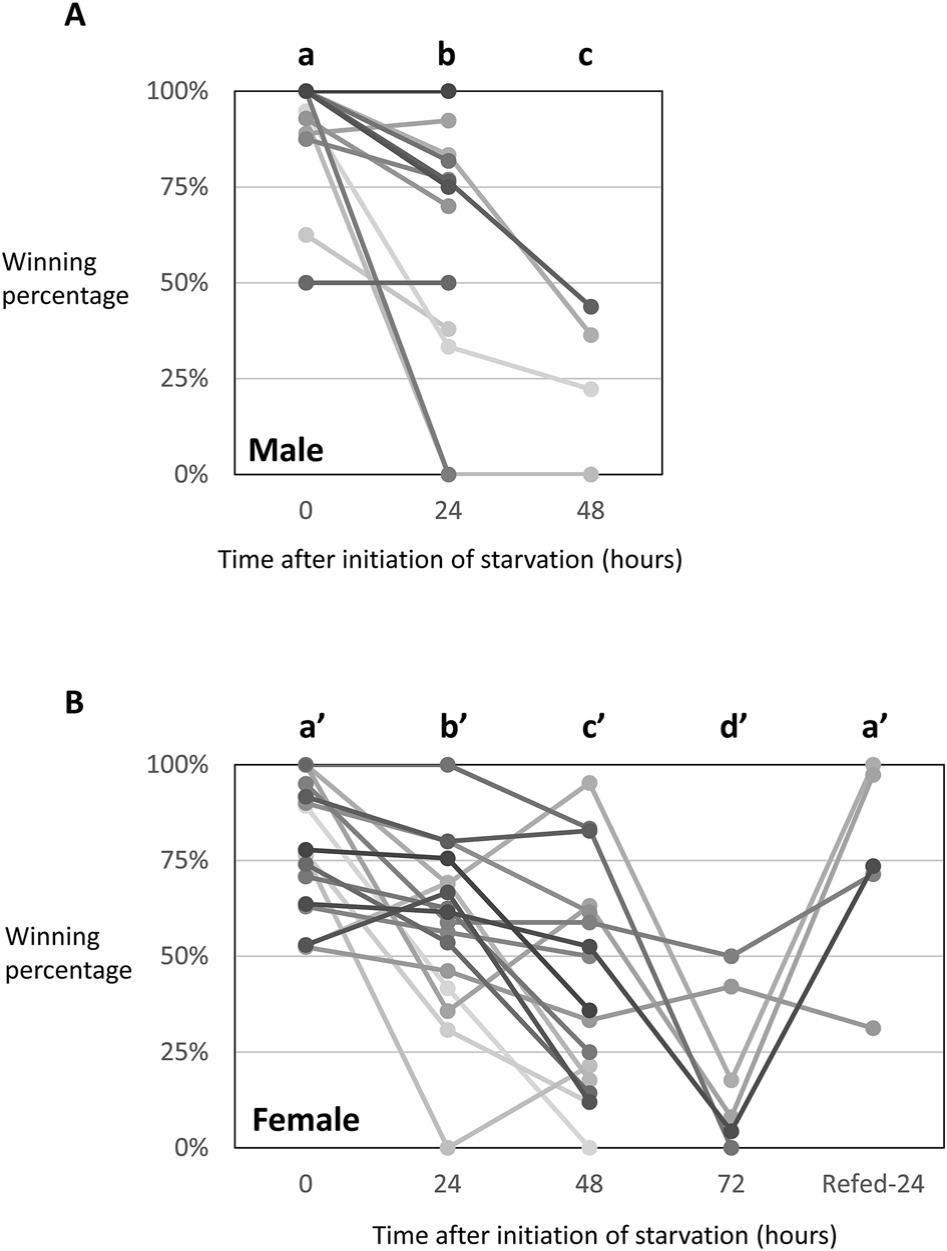
Starvation reduced the winning rate in *S. detrahens*. Matched and paired males (**A**) or females (**B**) were exposed to each other multiple times, and the winning rate in each contest was recorded (0 h). Then, the winners were starved, and the losers were continuously fed. At 24-h intervals after the initiation of starvation, the winning rates in the same pairs were measured. Fluctuations in the winning rates of the initial winner are shown by the single solid lines in the graphs. Breaks in the lines mean that measurement was not possible mostly because of death due to starvation. In the female pairs, at 72 h after the of initiation starvation, the initial winners were fed again to test the recovery of the winning rate, which was measured 24 h later. In each graph, time points labeled with different letters on the top (a, b, and c in graph (**A**) and a′, b′, c′ and d′ in graph (**B**)) indicate significantly different winning percentages (*P* < 0.05 after correction for multiple comparisons using the false discovery rate). The following analyses were conducted using R software version 4.0.2^59^.

Previous research has found that nutritional conditions in the larval stage influence the outcome of contests unless the ingested energy is not exhausted^43^. However, as mentioned above, the eye span of *C. dalmanni* is unlikely to be a signal of current energy retention. Furthermore, insect external organs that are covered with a hard exoskeleton, such as the eye stalk, cannot change in size even if the nutritional status changes after becoming an adult. Therefore, eye span cannot represent current energy stores exactly, even in the case of *S. detrahens,* in which eye span and starvation tolerance showed a strong positive correlation. Accordingly, we investigated whether the rate of victories or defeats between the same two individuals would change after starvation of the initial winner. In both *S. detrahens* and *C. dalmanni*, it has been reported that contests between males with similar eye spans do not end quickly^20,23^ and are not influenced by eye span. In such a situation, factors other than eye span will have a strong effect on the contest outcome. Therefore, we prompted two same-sex individuals with very close eye spans to compete and analyzed how the initial winner’s winning rate changed after 24, 48, and 72 h of starvation initiation in 13 male pairs and 17 female pairs, excluding pairs in which an individual died. A highly significant effect of the starvation (and refeeding) treatments was detected for both male and female *S. detrahens* (GLMM; *Χ*^2^_2_ = 93.6 and *Χ*^2^_4_ = 198.2, respectively, both *P* < 10^−15^). Although the average winning rate of the initial winners can be decreased to 50% in the second contest if winning randomly occurs, the winning percentage of the initial winner significantly and consistently decreased with the starvation period in both the males and females (Fig. 4A,B: average winning percentages of the initial winner, males = 87% [at 0 h], 60% [at 24 h], 26% [at 48 h]; females = 79% [at 0 h], 58% [at 24 h], 42% [at 48 h], and 20% [at 72 h]). Although most males died within 72 h of the onset of starvation, females remained partially viable. When we fed the surviving females exposed to starvation for 72 h and measured their winning percentages 24 h later, their winning rates recovered to a level comparable to those before starvation (Fig. 4B). These results suggest that the winning percentage between *S. detrahens* individuals with similar eye spans was affected by energy stores at the time of the contest.

## Discussion

### Starvation-induced autophagy in the fat body cells of the eye stalk

The eye stalks of *S. detrahens* had an approximately comparable amount of fat body tissue as the abdomen; in contrast, the eye stalks of *C. dalmanni* had only a small amount of fat body tissue relative to that in the abdomen. The fat bodies of insects often accumulate in the abdomen due to its expandable structure, but they do not always show an even distribution in the abdominal cavity. Unlike vertebrates, insects do not have blood vessels for rapid and active blood flow; therefore, fat bodies generally accumulate around organs that will require a large amount of energy in the future, such as the ovaries and digestive tract^39,44,45^. However, it is not thought that eye stalk fat body tissue in *S. detrahens* nourishes neighboring organs such as the brain and compound eyes. In fact, there is no reason why *S. detrahens* would require more energy in the brain and compound eyes than *C. dalmanni*. Furthermore, the rate of increase in autophagosomes/autolysosomes in fat body cells during starvation seemed to be slightly slower in the abdomen than in the eye stalks after 72 h of starvation, but there was no obvious difference between either location, suggesting that similar roles are played by the fat bodies in both locations in terms of starvation resistance. The eye stalk fat body in *S. detrahens* is thought to support starvation tolerance, much like the fat body cells distributed throughout the bodies of various insects^40,46,47^. This is supported by the strong correlation between eye span and starvation resistance in *S. detrahens*. In contrast, the fat body in the head of *C. dalmanni* occurs only around the brain (Fig. 1F); therefore, its energy supply might be directed to a limited extent, under normal nutrient intake conditions.

### Qualitative differences in fighting behaviors between the two species considering the cheaters and reliable signalers

There are conflicting interpretations of the meaning of signals displayed by individuals with exaggerated traits toward conspecifics. The Fisherian runaway hypothesis suggests that an exaggerated trait does not necessarily reflect the quality of the individual but provides benefits by being preferred by the opposite sex^48,49^. The handicap theory, on the other hand, suggests that the signal represents the quality of the individual since the growth of large traits requires sufficient energy retention, which is consistent with the theory of natural selection^1,3–5,7^. In fact, it is difficult to find an example case that can be explained solely by the runaway hypothesis, and the handicap theory is widely supported in the results of many behavioral studies^7^. In the case of courtship behavior, the signals sent to the opposite sex are considered to represent the information for holding good genetic quality in the signaler, but in the case of intra-sexual competition, it is reasonable to consider that the signals sent to rivals are to represent the energy storage ability. Then, the significance in the signals by the eye span is considered to be different between courtship and contest although both should correlate to various degrees and may not be independent. Unlike female *C. dalmanni*, female *S. detrahens* do not choose males according to their eye span; rather, males who have a long eye span tend to gain territory with a good food supply by winning contests and thus have an increased chance of mating with females, and both sexes tend to have high fertility when their eye spans are large^23^. A similar relationship between eye span and fertility in both sexes has also been shown in *C. dalmanni*^29–31^. These results suggest that the handicap theory can be applied to stalk-eyed flies as a whole reproductive population. However, the observed discordance between the eye spans and actual current energy stores (stamina) clearly indicates that some individuals can behave as cheaters in intra-sexual competition (male-male or female-female contests), piggybacking on the handicap mechanism to increase their fitness. The general and spontaneous occurrence of cheaters is theoretically predicted to be a real-life situation^5,50,51^, but in this study, it was recognized in the comparison between the two stalk-eyed fly species. The eye span in *S. detrahens* has the potential to signal an individual’s energy storage status to its opponent fairly accurately; however, this is not the case for *C. dalmanni*. This can be interpreted as the former conforming relatively well to the handicap theory, while the evolution of the latter may also include runaway processes via female choice. In other words, in ritualized contests, the winning rate in *C. dalmanni* does not reflect the amount of energy that can be invested in the altercation, and it is predicted that a large proportion of individuals who do not conform to the reliable signal system will win by bluffing. A previous report found that some genetic variants in *C. dalmanni* resulted in large eye spans regardless of larval nutrition, which may also be consistent with this expectation^32–34^.

### Difference in the significance of the eye span signal between the two species of stalk-eyed flies

The difference in mating behaviors between *C. dalmanni* and *S. detrahens* may contribute to the abundance of cheaters. As mentioned previously, *S. detrahens* females do not choose males according to eye span; rather, males choose individual females. Therefore, males do not form harems, and the energy currently possessed by males determines the number of consecutive matings in which they engage^23^. On the other hand, since the territory of an individual is small, even a male who loses a contest may get a new chance to mate immediately after the defeat. In contrast, in *C. dalmanni*, females actively choose males with long eye stalks, and males that have very long eye stalks form harems to ensure numerous mating opportunities^21,22^. Therefore, in terms of mating behavior, winning a territorial competition with another male is of great benefit, even if victory is achieved only once. On the other hand, due to the nature of harems, defeated males lose the opportunity to encounter many females, which is a great disadvantage. For this reason, it is conceivable that in *C. dalmanni*, there is greater value in investing energy in eye stalk extension during the pupal stage than in storing energy accumulated in the larval stage until the adult stage considering the chance of future harem formation. In fact, in this species, body length has a weak correlation with starvation tolerance, and it has been inferred that the energy possessed in the pupal stage is invested in eye stalk formation. This may be supported by the fact that the amount of fat in the abdomens of adults varies greatly from individual to individual. The eye span of *C. dalmanni* shows visible sexual dimorphism, which is consistent with the observed tendency for exaggerated male traits in various polygynous species, although exceptions can be found^2,52–55^.

### Eye spans display the limit of energy that can be retained, leading to the presence of cheaters

Although eye span can be a major factor in winning contests in *S. detrahens*^23^, the outcomes of contests between individuals with similar eye spans (Fig. 4) have shown that a larger eye span is not always conducive to winning, and the outcome can be affected by current energy stores. However, even if an individual is well nourished, it is difficult for individuals with very short eye spans to win contests. Although the behaviors of flies with smaller eye spans may differ from those of individuals with standard eye spans^24,56^, the fact that eye span represents the maximum amount of energy that can be stored rather than actual current energy stores may be the ultimate cause of their defeat. If this prediction is correct, all individuals, not only those with small eye spans, display the maximum amount of energy they can possibly possess, and individuals who are not currently well nourished will inevitably become cheaters that deceive opponents to varying degrees to increase their chance of winning. Therefore, a certain proportion of cheaters should always be present in populations of various stalk-eyed fly species, which may cause the correlation between eye span and reproductive capacity to remain below a certain level. Furthermore, as shown in the present study on *S. detrahens* and *C. dalmanni*, the frequency of cheaters could evolve variably depending on the mating mode of each species. It would be assumed that the frequency of cheaters will increase if the possession of an exaggerated trait results in a greater reproductive benefit, such as those in harem mating systems. How is the proportion of cheaters among individuals conforming to the handicap theory generally determined? If the benefit (increased fitness) gained by a similar signal intensity differs between the two species, then the species that obtains a greater benefit will have more cheaters due to utility maximization. Because the great benefit of winning is worth the challenge, many individuals would participate in competitions even with frequent defeats. A computer simulation study showed that more individuals with low resource holding potential tend to be cheaters when the value (fitness benefit) of the resource is higher^50,51^. This result is consistent with the different mating systems of the two species: in the harem mating system of *C. dalmanni*, possession of an exaggerated trait can result in a greater reproductive benefit.

### What does the weak correlation between trait size and starvation tolerance in females mean?

A weak correlation between trait size and starvation tolerance in females was found in both species. In *C. dalmanni* females in particular, not even a weak correlation was observed. This may be related to the lack of correlation between eye span and winning percentage in *C. dalmanni* females^42,43^. Three possible causes for this finding are as follows. (1) The eye span of *C. dalmanni* females is a gender load characteristic that is passed on due to trait exaggeration in males, because females share the same genes as males. The finding that many genes with sex-biased expression in eye imaginal discs are translocated to the sex chromosome^57^ and positive correlations between male and female eye spans among various species of stalk-eyed flies may be related to this phenomenon^13^. However, as many Diopsidae species show sexual monomorphy with regards to eye span^13–18^, their eye stalks are considered to have functions for other than contest and courtship even in females. (2) Females with long eye stalks are preferred by males, but long eye stalks induce a smaller benefit to females than to males, so they have gradually stopped evolving. (3) Female nutrition is mainly invested in promoting oogenesis, so energy allocation to contests is not high. There are few reasons to dispute these causes, so it is predicted that, in reality, these phenomena may explain the complex reasons for weak correlation between eye span and starvation tolerance in females.

## Methods

### Husbandry of *S. detrahens* and *C. dalmanni*

Wild individuals of *S. detrahens* were captured near the shores of several rivers on Ishigaki Island in Okinawa Prefecture (Japan) for several years after 2017 as described^58^. Wild individuals of *C. dalmanni* were captured in a tropical rainforest in the eastern area of Langkawi Island (Malaysia) in April 2018. Rearing methods for both species of stalk-eyed flies have been described previously^23^. In brief, dozens of adult individuals were maintained in large plastic dishes (245 × 245 × 25 mm, Nunc Inc. #240835) with supplies of water, commercially available semidried *Ficus* fruits (for adult provision), and royal jelly (for adult and larval provision). Numerous pin holes were bored into the lids of the dishes for aeration. The royal jelly was served on dried *Sphagnum* leaves (product of New Zealand, Nissin Garden Mate Co., #500) soaked with 20% (v/v) undiluted royal jelly supplemented with 0.05% (w/v) butyl parahydroxybenzoate (Antimold). To move adult flies in and out of the plastic dishes, the flies were exposed to carbon dioxide for a short period (less than one minute), resulting in anaesthetization.

### Microscopic examination of fat bodies stained with DAPI/phalloidin, oil red O, or LysoTracker

The fat bodies in the eye stalks and abdomen were dissected and fixed with 4% formaldehyde in phosphate buffered saline (PBS) for 20 min at room temperature. Then, the formaldehyde was removed by washing with PBS several times.

To visualize the distribution of the fat bodies in the eye stalks and the morphology of their cells, rhodamine-conjugated phalloidin (Molecular Probe, 1:200 dilution) was used to stain filamentous actin (-associated plasma membrane), and 4′6-diamidino-2-phenyl indole (DAPI, Sigma, 0.1 µg/ml) was used to stain nuclei.

To visualize neutral triglycerides and lipids in the lipid droplets, fixed fat bodies were soaked in 0.2% oil red O in 60% isopropanol for 20 min at room temperature and then washed with PBS.

To visualize lysosomes and autolysosomes, which increase during autophagy, unfixed fat bodies were soaked in a solution of LysoTracker Red (DND-99, Molecular Probes, 1:100 dilution) for 15 min at room temperature and then washed with PBS. Simultaneously, nuclei were counterstained with Hoechst 33258 (Molecular Probe, 1:500 dilution).

The stained specimens were observed under a VHX-2000 digital microscope (Keyence) and a C1Si (Nikon) or FV3000 (Olympus) laser confocal microscope. We also used the differential interference imaging system associated with the FV3000 system for the observation of lipid droplets. Quantification of LysoTracker-positive puncta per cell was performed by calculating the number of puncta in the 3D reconstitutions of the laser confocal images of fat bodies divided by the number of nuclei counterstained with Hoechst 33258. The value per nucleus was doubled to convert to the value per cell because the adult fat body cells are binucleate (Fig. 1B).

### Transmission electron microscopy (TEM)

Fat bodies obtained by dissection, as described above, were fixed with 2% glutaraldehyde/0.1 M phosphate buffer (pH 7.4) at 4 °C overnight. After washing with 0.1 M phosphate buffer three times, the tissues were subsequently fixed with 2% OsO_4_/0.1 M phosphate buffer. After fixation, a small piece of tissue was embedded in Quetol-812 resin (Nisshin-EM), and ultrathin sectioning (70-nm thickness) was performed; the sections were stained with 2% uranyl acetate for 15 min at room temperature followed by washing with H_2_O and secondary staining with lead stain solution (Signa-Aldrich) for 3 min at room temperature. TEM photographs were obtained using a JEM-1400Plus instrument (JEOL) with an accelerating voltage of 100 kV.

### Measurement of eye span length

By using a VHX-2000 digital microscope (Keyence), eye span lengths were quantified as the linear distance between the left and right outer edges of the compound eyes. Although each eye span was measured only once, measurement error could be neglected because the relative standard error of eye span was 0.35% when a representative sample was measured 20 times in various possible positions.

### Quantification of starvation tolerance

Fully fed individuals were transferred into individual transparent plastic vials for *Drosophila* culture (Hightech Co., 9.5 cm × ϕ2.2 cm), which comprised a solidified mixture of 5 ml of 1% agar (S-6, Ina Food Co., Ltd.) in deionized water. Multiple vials were aligned in an upright position and kept at 25 °C, and a RICOH GXR digital camera captured time-lapsed images of the side view of the vials in 10-min intervals for several days. When a fly fell to the surface of the agar and did not move thereafter, it was considered dead, and the time was recorded. Calculation of the correlation coefficient (*r*) and *P* values for the relationship between eye span/body length and starvation tolerance, in addition to linear approximation with Model 1 (OLS) regression, was carried out in Microsoft Excel ver.16.0.15128.20248 (Microsoft Corporation, https://microsoft-excel.en.softonic.com/).

### Observation of contests between fed and starved *S. detrahens* individuals with similar eye spans

Dozens of individuals with a moderate eye span were selected, and individual numbers were assigned to males and females separately, with the lowest number assigned to the individual with the shortest eye span. Males and females with consecutive numbers representing similar eye spans were defined as a pair to be contested. All the actual ratios of the eye spans between two matched individuals were less than 1.03. This ratio was established because the effect of starvation was more apparent when the difference in the eye span between two individuals was small, resulting in a winning probability of approximately 0.5, especially for males^23^. Two matched flies (male vs. male or female vs. female) were placed in a Petri dish after being anesthetized with carbon dioxide gas. After the flies were sufficiently awake, videos were recorded by a JVC Everio R video camera for 2 h. The cessation of locomotion in both individuals in a face-to-face orientation was defined as the beginning of a contest. Then, backwards or sideways movements were defined as the loser’s motions. When both individuals showed backwards motion at the same time, the contest was declared a draw. The winning percentage was calculated by dividing the number of wins by the number of competitions, including draws. After the initial contests, starvation of the winners was initiated, and the winning rates of the fixed pairs were measured at 24-h intervals. In the case of the females, at 72 h of starvation, the initial winners were fed, and their winning rates were calculated after an additional 24 h. The records of pairs in which one individual died within 48 h were excluded in the calculation of winning rates. Contests comprising fewer than 4 competitions were also excluded to avoid incidental fluctuations in the winning rate. The following analyses were conducted using R software version 4.0.2^59^. To analyze the effects of starvation on the winning rate (weighted by the number of competitions), generalized linear mixed models (GLMMs) were fitted using the “glmer()” function in the “lme4” package^60^ by incorporating contestant-pair identity as a random effect factor. A binomial error structure and a logistic link function were used. We examined the significance of starvation and refeeding treatments (a fixed effect factor) based on a likelihood ratio test between the full (with the fixed factor) and null (with only the random factor) models. When a significant treatment effect was detected, the same analysis was applied for post hoc pairwise multiple comparisons between starvation (and refeeding) time points. Significance thresholds in multiple comparisons were corrected using the false discovery rate^61^.
